# Low Long Noncoding RNA Growth Arrest-Specific Transcript 5 Expression in the Exosomes of Lung Cancer Cells Promotes Tumor Angiogenesis

**DOI:** 10.1155/2019/2476175

**Published:** 2019-05-02

**Authors:** Ying Cheng, Xin Dai, Ti Yang, Nan Zhang, Zhenzhong Liu, Yiguo Jiang

**Affiliations:** ^1^State Key Laboratory of Respiratory Disease, The First Affiliated Hospital of Guangzhou Medical University, 510120, China; ^2^Institute for Chemical Carcinogenesis, Guangzhou Medical University, Guangzhou 511436, China

## Abstract

Angiogenesis plays a key role in the development and progression of lung cancer. Recent studies have found that tumor cells can stimulate angiogenesis by secreting exosomes, which contain many long noncoding RNAs (lncRNAs), some of which are important for the development of lung cancer. However, the roles and mechanisms of exosomal lncRNAs in lung cancer angiogenesis have not yet been reported. In this study, lung cancer in mice was induced by urethane; we found that growth arrest specific 5 (GAS5) was lowly expressed in the serum exosomes and lung cancer tissues of mice with lung cancer. And there was a significant positive correlation between GAS5 expression in serum exosomes and lung cancer tissues. Furthermore, GAS5 was lowly expressed in human lung cancer tissues, lung cancer cells, and cells culture supernatant exosomes. The exosomes of lung cancer cells promoted human umbilical vein endothelial cells (HUVECs) proliferation and tube formation and inhibited their apoptosis. GAS5 overexpression in lung cancer cells increased GAS5 level in cell culture supernatant exosomes. And the exosomes of lung cancer cells containing high GAS5 level inhibited HUVECs proliferation and tube formation and increased their apoptosis. In addition, we found that GAS5 competitively bound miRNA-29-3p with phosphatase and tensin homolog (PTEN), upregulating PTEN mRNA and protein expression, and inhibited level of phosphatidylinositol-4,5-bisphosphate 3-kinase catalytic subunit alpha (PI3K) and serine/threonine kinase 1 (AKT) phosphorylation in HUVECs. Overall, our results suggest that exosomal GAS5 could be a new therapeutic target for lung cancer which inhibits angiogenesis.

## 1. Introduction

Lung cancer is one of the most common malignancies, and, despite improvements in the technologies used to treat this disease, its prognosis remains poor. Both the growth and the metastasis of lung cancer require support from newly formed vessels. It has been found that lung tumors can induce angiogenesis. Thus, inhibiting angiogenesis has become an important direction for lung cancer treatment [1].

Exosomes are a class of small (30–100 nm) vesicles that are released from a variety of living cells that contain specific proteins, lipids, and nucleic acids [2]. These substances can be transported to adjacent cells or distant cells via exosomes, functioning as novel form of intercellular information exchange. It has recently been found that exosomes participate in the communication between tumor cells and normal cells in the tumor microenvironment, playing diverse roles such as promoting tumor growth, metastasis, and angiogenesis [3–5].

Long noncoding RNAs (lncRNAs) are noncoding RNAs greater than 200 nt in length. lncRNAs play important roles in a variety of processes in cancer such as tumorigenesis, progression, angiogenesis, and metastasis [6, 7]. In addition to regulating gene expression by directly participating in DNA methylation, histone modification, and chromatin remodeling, lncRNAs can also act as competing endogenous RNAs (ceRNAs), which compete with other RNA transcripts to bind miRNAs, antagonizing their functions by reducing the silencing effect of these miRNAs on their target mRNAs [8–10]. Many functional lncRNAs are specifically enriched in exosomes, and whether these lncRNAs can be used as reliable markers for tumor diagnosis or as new targets for antitumor therapy is also being explored. To date, lung cancer-associated exosomal lncRNAs and their roles and underlying mechanisms in lung cancer angiogenesis have not been reported.

In the current study, using urethane-induced lung cancer mouse model, we screened some lncRNAs expression in the serum exosomes of mice and then we further investigated the functions and potential mechanisms of the exosomal lncRNA growth arrest specific 5 (GAS5), which is relatively highly homologous in human and mouse, in human lung cancer cells and human umbilical vein endothelial cells (HUVECs). Our study found that lung cancer-derived exosomal GAS5 affected the proliferation, apoptosis, and tube formation of HUVECs. GAS5 competitively binds to miRNA-29-3p with phosphatase and tensin homolog (PTEN) in HUVECs to upregulate the PTEN expression and inhibit the phosphorylation of PI3K/AKT, suggesting GAS5 plays an important role in lung cancer angiogenesis.

## 2. Materials and Methods

### 2.1. Cell Lines and Materials

16HBE cells were kindly donated by Professor Jun Xu of the Guangzhou Medical University State Key Laboratory of Respiratory Diseases [11]. A549, H1299, and 95D cells were purchased from the Chinese Academy of Sciences Type Culture Collections Cell Bank (Shanghai, China). The primary human umbilical vein endothelial cells (HUVECs) were purchased from Shanghai AllCells Biotechnology Company (Shanghai, China). These cells were cultured in RPMI 1640 medium (Thermo Fisher Scientific, Waltham, MA, USA) containing 10% fetal bovine serum (sijiqing, Guangzhou Weijia Co. Ltd., Guangzhou, China), 1% penicillin, and 1% streptomycin (Thermo Fisher Scientific). 16HBE cells were cultured in modified Eagle's medium (MEM) (Thermo Fisher Scientific) containing 10% fetal bovine serum (sijiqing), 1% penicillin, and 1% streptomycin (Invitrogen, Carlsbad, CA, USA). HUVECs cells were cultured in Endothelial Cell Medium (ECM) containing 5% fetal bovine serum, 1% penicillin, and 1% streptomycin (Sciencell, Carlsbad, CA, USA). All cell lines were maintained at 37°C in a humidified incubator (5% CO_2_).

### 2.2. Human Lung Cancer Specimens

Thirty nonsmall cell lung cancer tissue specimens and matched adjacent normal tissue specimens (more than 5 cm from cancerous tissue) were collected from patients who underwent radical or palliative resection in the Department of Thoracic Surgery, the First Affiliated Hospital of Guangzhou Medical University, between October 2015 and January 2016.

### 2.3. Animal Experiments

Ninety-six BALB/C mice (4–6 weeks old, same number of females and males) were purchased from the Guangdong Provincial Experimental Animal Center. They were fed for 3 weeks and then divided into the negative control and urethane induction groups. Mice in the urethane induction group were intraperitoneally injected with 1g/kg urethane (Sigma-Aldrich, St. Louis, MO, USA) once a week for 4 weeks, and the control mice were given same injections of normal saline. The mice were then fed for another 6, 9, or 12 weeks. Sixteen mice in each group were sacrificed; serum and lung tissues were collected. LncRNA expression analyses were performed on serum and tissues (n=12 per group) and pathological examination was performed on lung tissues (n=4 per group).

### 2.4. RNA Isolation and qRT-PCR Analysis

Exosome was isolated using the Total Exosome Isolation Reagent (from serum or cell culture media) (Invitrogen, Carlsbad, CA, USA). Morphology of exosome was analyzed using electron micrography. Concentration of exosome was measured using nanoparticle tracking analysis (NTA). Exosomal RNA was isolated using the Exosome RNA Extraction Kit (Invitrogen, Carlsbad, CA, USA). Briefly, we resuspended the exosomes, added Denaturing Solution, incubated the mixture on ice for 5 minutes, added Acid-Phenol Chloroform, mixed samples and then centrifuged for 5 minutes at 10,000 g at room temperature, removed the upper phase, added 1.25 volumes 100% ethanol to the tube and mixed thoroughly, pipetted the mixture onto the Filter Cartridge, centrifuged at 10,000 g for 15 seconds, discarded the flow-through, added Wash Solution 1, centrifuged at 10,000 g for 15 seconds, discarded the flow-through, added Wash Solution 2/3 and drew it through the filter, added 50 *μ*l of preheated Elution Solution, and centrifuged for 30 seconds to recover the RNA. Tissues or cellular RNA was isolated using the TRIzol (Invitrogen, Carlsbad, CA, USA). The nuclear or cytoplasmic RNA was isolated using the PARIS™ Kit (Ambion, Carlsbad, CA, USA). cDNAs were synthesized according to the manual of the Go Script™ Reverse Transcription System Kit, and PCR reactions were conducted on Applied Biosystems® 7500 Real-Time PCR Systems according to the manual of the GoTaq® qPCR Master Mix Kit (Promega, Madison, WI, USA). Primers used in the study are shown in Supplement Tables 1–3. cel-miR-39-3p was used as the external reference. GAPDH served as the internal reference, and the relative expression of cellular or tissue lncRNAs was calculated using the 2^−ΔΔCt^ method.

### 2.5. Cell Proliferation Assay

The proliferative rate of HUVECs was tested using the EdU assay (Guangzhou Ruibo Bioscience Co. Ltd., Guangzhou, China). Cells were seeded onto a 24-well plate and cultured in medium containing 1% fetal bovine serum for 24 h. Cells were then processed according to different study purposes. The cells were incubated with EdU at 37°C for 8 hours stained with Apollo for 30 min in the dark at room temperature. The cells were then incubated with Hoechst33342 at room temperature in the dark for 30 min. All nuclei were stained by Hoechst33342, which showed dark blue fluorescence at 350 nm excitation. EdU stained nuclei in S phase; these nuclei showed red fluorescence at 550 nm excitation.

### 2.6. Apoptosis Assay

The apoptosis rates of HUVECs were tested by Annexin V–FITC staining (Abcam, Cambridge, UK). Cells were seeded onto a 24-well plate and cultured in medium containing 1% fetal bovine serum for 24 h. The cells were then harvested and resuspended in 500 *μ*l phosphate buffered saline. Annexin V–FITC and PI were added and incubated in the dark at room temperature for 15 min. Apoptosis was measured on a FACS can flow cytometer.

Nuclear DNA breakage during early apoptosis was detected by the TUNEL assay (Roche, Basel, Switzerland). A cover slip was placed on the 24-well plate, and cells were seeded on the cover slip by overnight culture. The TUNEL reaction mixture was added and incubated in a dark humid chamber for 1 h. Converter-POD solution was added and incubated at 37°C for 30 min. DAB was added for 5 min, and hematoxylin was used as a counterstain by incubating for several seconds. After gradient alcohol dehydration, drying, and mounting with neutral balsam, the slide was observed under a microscope. Positive staining was identified as brown nuclei, and blue represented hematoxylin-counterstained nuclei.

### 2.7. Matrigel-Based Tube Formation Assay

HUVECs were seeded in a 6-well plate and starved in the medium containing 1% fetal bovine serum for 24 h. Matrigel was thawed at 4°C overnight, and pipette tip boxes, EP tubes, and 96-well plates were all precooled at 4°C. Matrigel gel was diluted with serum and growth factor free ECM medium (1:1), mixed evenly, and added into a 96-well plate (50 *μ*l/well). All procedures were performed on ice. The plate was placed in a 37°C incubator for 2 h. Trypsin was used to digest HUVECs, which were then seeded in the Matrigel-prepared 96-well plate (10,000 cells per well) and cultured for 4 h. The cells were observed under a microscope, and Image-Pro Plus was used to process tube formation images to calculate the total length of the tubes.

### 2.8. Lentiviral-Mediated GAS5 Overexpression

Recombinant shuttle plasmids and packaging plasmids including pGag/Pol, pRev, and pVSV-G were constructed by Shanghai GenePharma Co. Ltd. Viral packaging, collection, and titer testing were also conducted by the company. The recombinant shuttle plasmids were fifth-generation Lentivirus 5 particles. The constructed lentiviral vectors included LV5-NC (negative control) and LV5-GAS5 (GAS5 overexpression). Expression experiments were performed according to the company's manual. Briefly, cells were seeded in a 6-well plate 1 d before the experiment. Virus infection was performed when cultured cells grew to 40%–60% confluence. The viral master solution was diluted 10 folds and added to the medium. After 8–12 h incubation, the status of the cells was observed, and there was no significant difference between control and infected cells. The cells were cultured, and the culture medium was replaced after 24 h. After 48 h viral infection, GAS5 expression was assessed by observing fluorescence signal. Puromycin was used to select stable strains.

### 2.9. Luciferase Reporter Assay

Sequence comparisons were performed for GAS5, PTEN and miR-29a-3p, miR-29b-3p, and miR-29c-3p. pmiRGlo-GAS5 and pmiRGlo-PTEN-3′UTR, which were dual luciferase reporter vectors that incorporated miR-29a-3p, miR-29b-3p, and miR-29c-3p binding sites, were constructed. The vectors were cotransfected with miR-29a-3p, miR-29b-3p, and miR-29c-3p mimics for 48 h, and then luciferase activity was tested according to the manual from the kit (Promega). The activities of the reporter genes were standardized to Renilla luciferase activity (internal control).

### 2.10. Transfection

Cells were transfected according to the Lipofectamine™ 3000 protocol (Invitrogen). One day before transfection, cells were digested with trypsin, counted, and seeded at 70%–90% confluency for transfection. For each well, 50 *μ*l of serum-free medium was used to dilute miR-29-3p mimics and Lipofectamine™ 3000 reagent, which were mixed and incubated at room temperature for 20 min. The transfection mixture was directly added to each well and mixed by shaking. The medium was changed after 6 h, and transfection continued for 48 h.

### 2.11. Western Blot

According to the instructions of the exosome protein extraction kit (Invitrogen, Carlsbad, CA, USA), isolated exosomes were mixed with a five times Laemmli Sample Buffer (10%  *β* mercaptoethanol, Bio-Rad, USA), diluted with PBS to acquire an equal concentration of protein. Protein lysis buffer containing PMSF, protease, and phosphatase inhibitors was used to extract total proteins. The target proteins and internal reference protein were separated under the indication of prestained marker proteins, followed by membrane transfer and incubation with specific primary antibodies at 4°C overnight. The antibodies used include Rabbit anti-CD34, anti-CD63 and anti-GAPDH (Abcam), rabbit anti-PTEN, anti-PI3K, anti-p-PI3K, anti-AKT, and anti-p-AKT (Affinity, Cincinnati, OH, USA). Signals were detected using secondary antibodies labeled with IRDye 800 (Rockland Immunochemicals, Gilbertsville, PA) and signal intensities were determined using the Odyssey Infrared Imaging System (LI-COR Biosciences, Lincoln, NE, USA).

### 2.12. Statistical Analysis

Date were analyzed using GraphPad Prism 5 and SPSS17.0 software. Data were expressed as mean ± SEM. Statistical analysis was performed by independent sample t-test, one-way ANOVA, and univariate linear regression, and* P <*0.05 was considered indicative of statistical significance.

## 3. Results

### 3.1. Low GAS5 Levels in Serum Exosomes and Lung Cancer Tissues of Mice with Lung Cancer

**Figure 1:**
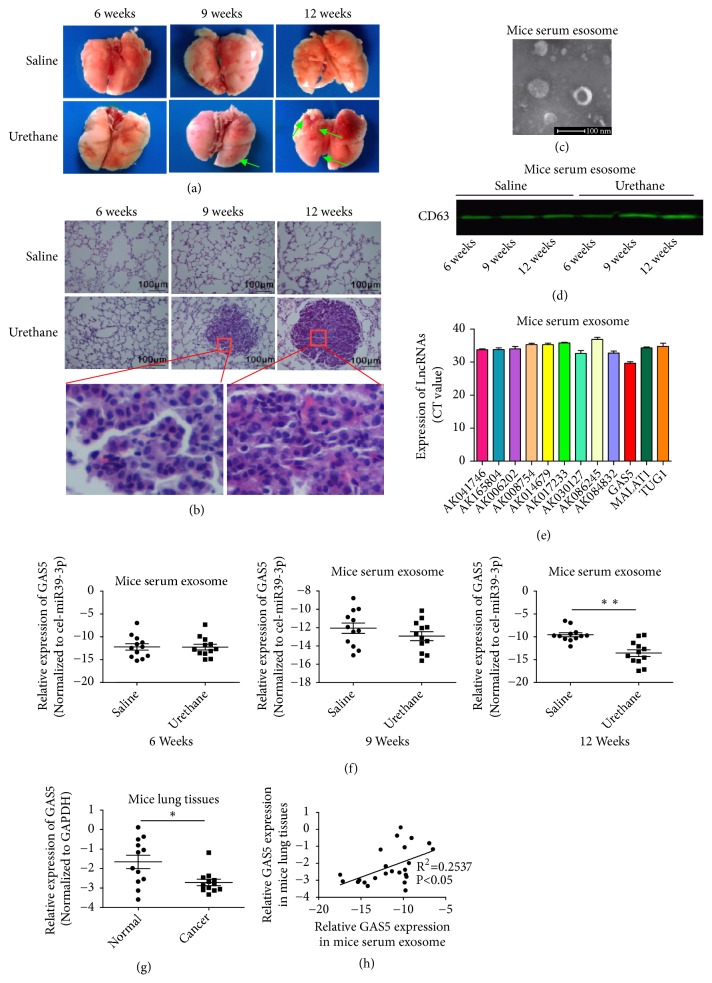
*Low GAS5 level in serum exosomes and lung cancer tissues of mice with lung cancer.* (a) Gross anatomy of the lungs from normal saline control and urethane-treated mice at different time points. (b) Representative pathological images of the lungs from control and urethane-treated mice at different time points (200×magnification, except the images from the 9th and 12th weeks which were 400×magnification). (c) Representative images of isolated exosomes by electron microscopy. (d) Exosome marker CD63 was detected by western blot. (e) lncRNAs level in mouse serum exosomes were detected by qPCR. (f) GAS5 level in serum exosomes was detected by qPCR. cel-miR-39-3p served as the external reference. (g) GAS5 level in the lungs of control and urethane-treated mice in the 12th week. (h) Correlation between serum exosomal GAS5 level and GAS5 expression in the lungs of mice was analyzed by univariate linear regression. Statistical significance, *∗P*<0.05, *∗∗P*<0.01.

We used urethane to induce lung cancer in mice, and, 9 and 12 weeks after urethane treatment, we observed tumor formation on the lung surface of treated mice (Figure 1(a)). In the 9th week, 6 out of 16 mice showed tumor formation. In the 12th week, all 16 mice showed tumor formation and there were a total of 53 tumors. Pathological examination confirmed that these urethane-induced tumors were intermediate or poorly differentiated lung adenocarcinomas (Figure 1(b)). We extracted mouse serum exosomes via serum exosome extraction kit, isolated particles that presented bona fide characteristics of exosomes, i.e., cup-shaped appearance in electron microscopy (Figure 1(c)). Then the specific exosomal marker CD63 was detected by western blot. The results showed that CD63 was enriched in the extracted granule precipitation (Figure 1(d)), suggesting exosomes with good quality were obtained. By analyzing our published microarray data from urethane-induced lung cancer in mice and reviewing recent literature regarding lung cancer-associated lncRNAs, we selected 19 lncRNAs including AK016354, AK041746, AK169506, AK165804, AK006202, AK008754, AK014679, AK017233, AK030127, AK086245, AK040806, AK084832, MALAT1, HOTAIR, GAS5, LINCRNA-P21, SOX2OT, SNHG1, and TUG1 for further analysis. Real-time qPCR was used to detect the levels of these lncRNAs in mouse serum exosomes, among which, 12 were stably detected (Figure 1(e)). The CT value of one lncRNA, GAS5, was more stable and smaller than the other lncRNAs, indicating the lncRNA was consistently expressed and stably present in mouse serum exosomes. The current literature reports that GAS5 is an important molecule for the proliferation, apoptosis, invasion, metastasis, and angiogenesis of lung cancer. Therefore, we further detected GAS5 levels in serum exosomes of mice by qPCR. The results showed that, at week 12, GAS5 levels in serum exosomes from urethane-induced lung cancer mice were significantly lower than those in the control group (Figure 1(f)). GAS5 expression in normal lung tissue and lung cancer tissue was also detected by qPCR. The results showed GAS5 expression in lung cancer tissues of urethane-treated mice was significantly lower than that in normal lung tissues (Figure 1(g)). Univariate linear regression supported a significant positive correlation between GAS5 levels in serum exosomes and GAS5 expression in lung tissues at week 12 (Figure 1(h)).

### 3.2. Low GAS5 Expression in Tissues, Cells, and Cell-Derived Exosomes of Human Lung Cancer

**Figure 2:**
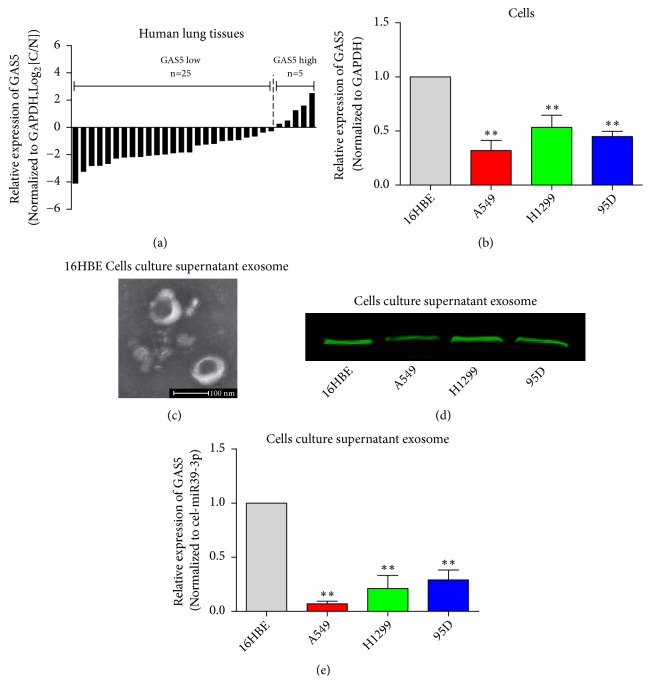
*Low GAS5 expression in tissues, cells, and cell-derived exosomes of human lung cancer.* (a) GAS5 expression in human lung cancer tissues and paracancerous tissues was detected by qPCR; GAPDH served as the internal reference. (b) GAS5 expression in human lung cancer cells was detected by qPCR; GAPDH served as the internal reference. (c) Representative images of isolated exosomes by electron microscopy. (d) Exosome marker CD63 was detected by western blot. (e) The GAS5 level of cell culture supernatant exosomes from lung cancer cell lines, cel-miR-39-3p served as the external reference. Statistical significance, *∗P*<0.05, *∗∗P*<0.01.

GAS5 expression was next detected by qPCR in human lung cancer tissues, the lung cancer cell lines (A549, H1299, 95D), and normal bronchial epithelial cells (16HBE). The results showed that in 30 pairs of matched lung cancer samples (cancerous vs. adjacent normal tissues), there were 25 cases showing lower GAS5 expression in cancer tissues than adjacent normal tissues (Figure 2(a)). Furthermore, GAS5 expression was significantly lower in A549, H1299, and 95D than 16HBE cells (Figure 2(b)). Isolated particles presented bona fide characteristics of exosomes, i.e., cup-shaped appearance in electron microscopy (Figure 1(c)). To demonstrate that granule precipitates contained high CD63 levels, the exosomal marker protein was detected in cell culture supernatant exosomes by western blot (Figure 2(d)). These data suggested high-quality exosomes were obtained. qPCR showed that GAS5 levels in the cell culture supernatant exosomes of A549, H1299, and 95D cells were significantly lower than 16HBE cells (Figure 2(e)).

### 3.3. Lung Cancer Cell-Derived Exosomes Promoted HUVECs Proliferation and Tube Formation and Inhibited Their Apoptosis

**Figure 3:**
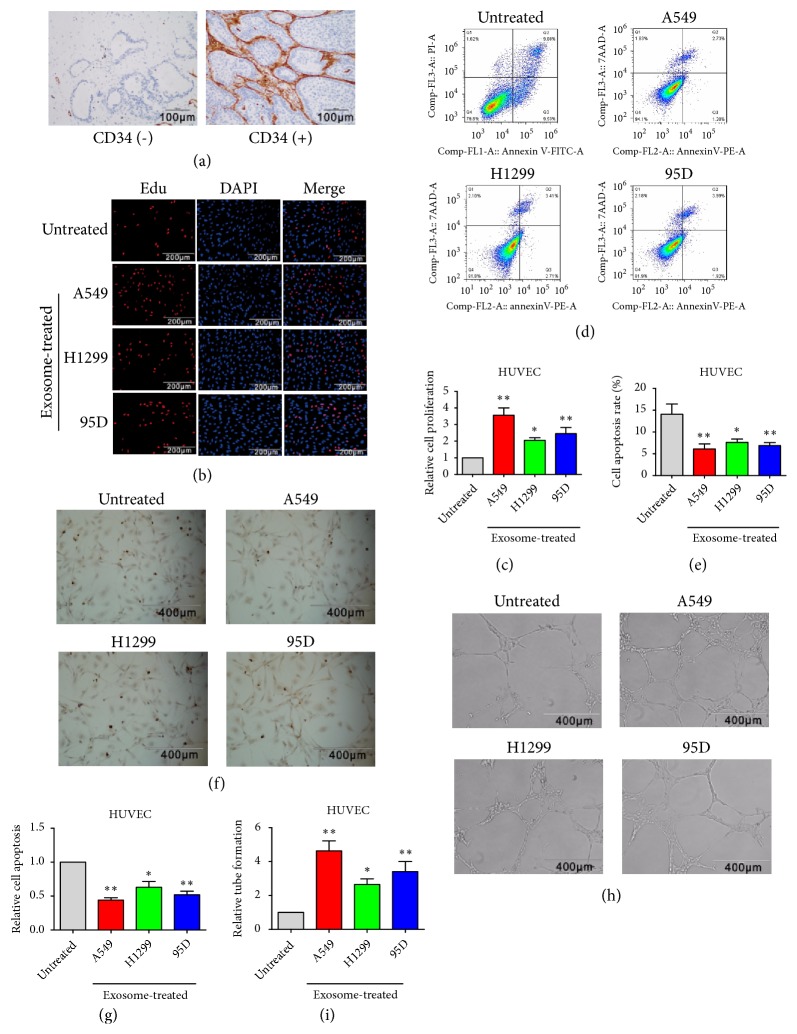
*Lung cancer cell-derived exosomes promoted HUVECs proliferation and tube formation and inhibited their apoptosis.* (a) CD34 expression in human lung cancer tissues was detected by immunohistochemistry. (b, c) After 48 h treatment with cell culture supernatant exosomes from different lung cancer cells, the proliferation of HUVECs was detected by the EdU assay. HUVECs without exosome treatment were used as control group. (d, e) Apoptosis in HUVECs was detected by FCM. (f, g) Apoptosis in HUVECs was detected by the TUNEL assay. (h, i) The tube formation capacity of HUVECs was assessed by the 3D Matrigel assay. Statistical significance, *∗P*<0.05, *∗∗P*<0.01.

CD34 was used to measure lung cancer angiogenesis. Immunohistochemistry showed positive CD34 staining in 83.3% of the 30 lung cancer tissue samples, indicating angiogenesis is common in lung cancer (negative and positive results are shown in Figure 3(a)). About 8×10^6^ particles/ml of exosomes collected from cell culture supernatant of the lung cancer cell lines A549, H1299, and 95D after 48 h incubation were then used to treat human umbilical vein endothelial cells (HUVECs) for 48 h, following which the HUVECs proliferation was detected by the EdU assay. Furthermore, apoptosis and tube formation of HUVECs were measured by FCM and TUNEL, and 3D Matrigel assays, respectively. EdU results showed that, compared with the control group, exosomes from A549, H1299, and 95D cells significantly increased HUVECs proliferation (Figures 3(b) and 3(c)). FCM showed that cell culture supernatant exosomes from A549, H1299, and 95D cells significantly inhibited HUVECs apoptosis (Figures 3(d) and 3(e)). The TUNEL assay corroborated the FCM results (Figures 3(f) and 3(g)). 3D Matrigel assays showed exosomes from A549, H1299, and 95D cells significantly enhanced tube formation in HUVECs (Figures 3(h) and 3(i)).

### 3.4. GAS5 Overexpression Reversed the Effects of Lung Cancer Cell-Derived Exosomes on HUVECs

**Figure 4:**
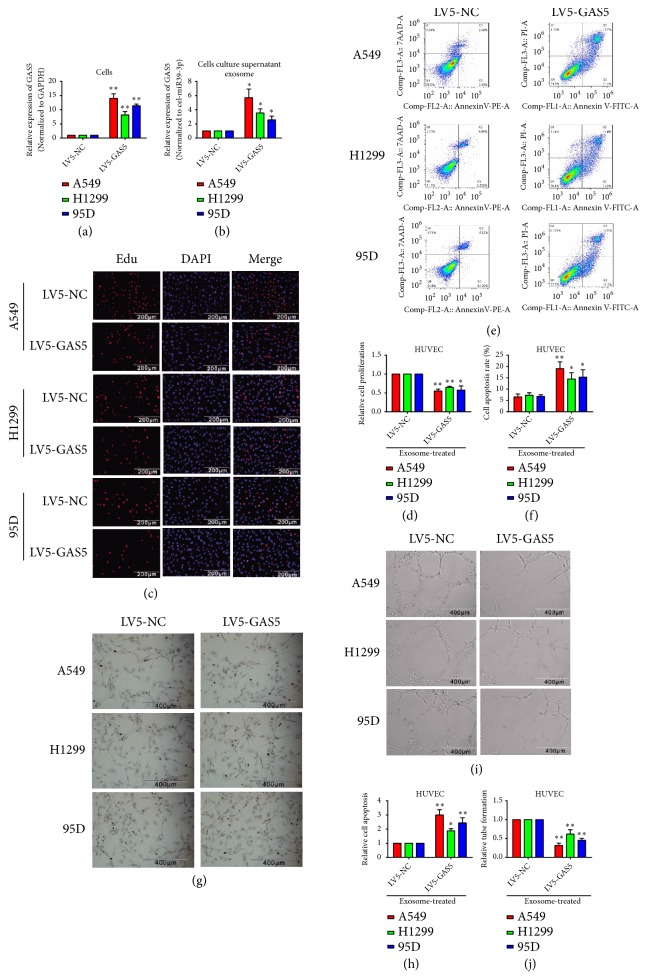
*GAS5 overexpression reverses the effects of lung cancer cell-derived exosomes on HUVECs.* (a) GAS5 expression in lung cancer cells, in which GAS5 was overexpressed via lentiviral transduction, was detected by qPCR; GAPDH served as the internal reference; LV5-NC was used as control group. (b) GAS5 level in the cell culture supernatant exosomes was detected by qPCR, cel-miR-39-3p served as the external reference. (c, d) After 48 h treatment with the cell culture supernatant exosomes from control or GAS5-overexpressing lung cancer cells, the proliferation of HUVECs was detected by the EdU assay. (e, f) The apoptosis of HUVECs was detected by FCM. (g, h) The apoptosis of HUVECs was detected by the TUNEL assay. (i, j) The tube formation capacity of HUVECs was assessed by the 3D Matrigel assay. Statistical significance, *∗P*<0.05, *∗∗P*<0.01.

GAS5 overexpression vectors were constructed based on the fifth-generation lentivirus 5. The control or overexpression groups were represented as LV5-NC or LV5-GAS5. After overexpressing GAS5 in the lung cancer cell lines A549, H1299, and 95D, GAS5 levels were significantly increased (Figure 4(a)). Cell culture supernatant exosomes from the control or GAS5-overexpressing groups were collected after 48 h culture, and qPCR was used to detect GAS5 levels. Compared with the control group, GAS5 levels were significantly increased in the overexpression groups (Figure 4(b)). About 8×10^6^ particles/ml of exosomes from the control or GAS5 overexpression cells culture supernatant were then used to treat HUVECs for 48 h; HUVECs proliferation, apoptosis, and tube formation were measured by the respective assays. EdU assays showed that exosomes from GAS5 overexpression cells inhibited HUVECs proliferation compared with control exosomes (Figures 4(c) and 4(d)). FCM showed that exosomes from GAS5 overexpression cells enhanced HUVECs apoptosis (Figures 4(e) and 4(f)). TUNEL assays showed similar results as FCM (Figures 4(g) and 4(h)). 3D Matrigel assays showed that exosomes from GAS5 overexpression cells inhibited HUVEC tube formation (Figures 4(i) and 4(j)).

### 3.5. GAS5 Regulated PTEN Expression Affected PI3K and AKT Phosphorylation in HUVECs

**Figure 5:**
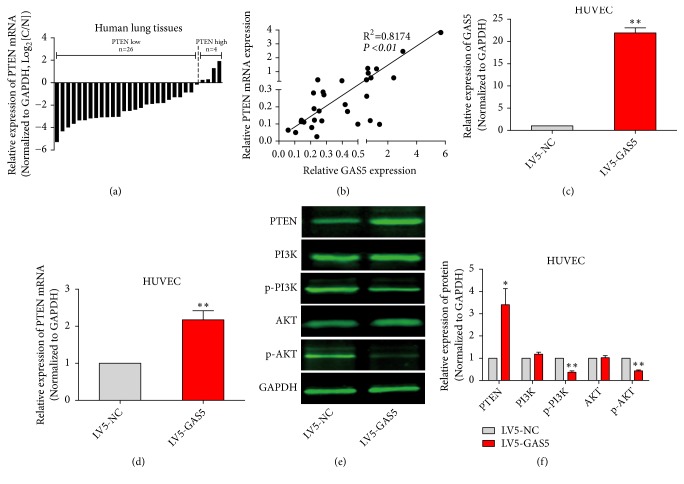
*GAS5 regulated PTEN expression and affected PI3K and AKT phosphorylation in HUVECs.* (a) PTEN mRNA expression in human lung cancer tissues and adjacent normal tissues was detected by qPCR; GAPDH served as the internal reference. (b) The linear correlation between PTEN mRNA and GAS5 expression in human lung cancer tissues was analyzed by univariate linear regression. (c) GAS5 expression in HUVECs, in which GAS5 was overexpressed via lentiviral transduction, was detected by qPCR; GAPDH served as the internal reference. LV5-NC was used as control group. (d) The level of PTEN mRNA expression in HUVECs detected by qPCR, GAPDH served as the internal reference. (e, f) Total PTEN, PI3K, and AKT and phosphorylated p-PI3K and p-AKT levels were detected by western blot. Statistical significance, *∗P*<0.05, *∗∗P*<0.01.

PTEN is important for lung cancer angiogenesis. Thus, we next detected PTEN mRNA expression in human lung cancer and normal lung tissues by qPCR, finding that PTEN expression was significantly decreased in cancer tissues relative to adjacent lung tissues. Among 30 paired lung cancer samples, 26 cases showed lower PTEN mRNA expression in cancer tissues than adjacent lung tissues (Figure 5(a)). Univariate linear regression showed a significant positive correlation between PTEN and GAS5 expression (Figure 5(b)). We then overexpressed GAS5 in HUVECs (Figure 5(c)). At the same time, PTEN mRNA levels in the overexpression group also were significantly increased (Figure 5(d)). The expression of PTEN protein was detected by western blot. PTEN protein expression in the overexpression group increased significantly. PTEN is a tumor suppressor gene with phospholipase activity that is involved in regulating the phosphorylation of PI3K and AKT. Therefore, we next examined PI3K and AKT protein levels and levels of their phosphorylation (p-PI3K and p-AKT). Levels of PI3K and AKT showed no significant change, while p-PI3K and p-AKT decreased significantly (Figures 5(e) and 5(f)).

### 3.6. GAS5 Regulated PTEN, PI3K, and AKT by Competitively Binding miR-29-3p with PTEN

**Figure 6:**
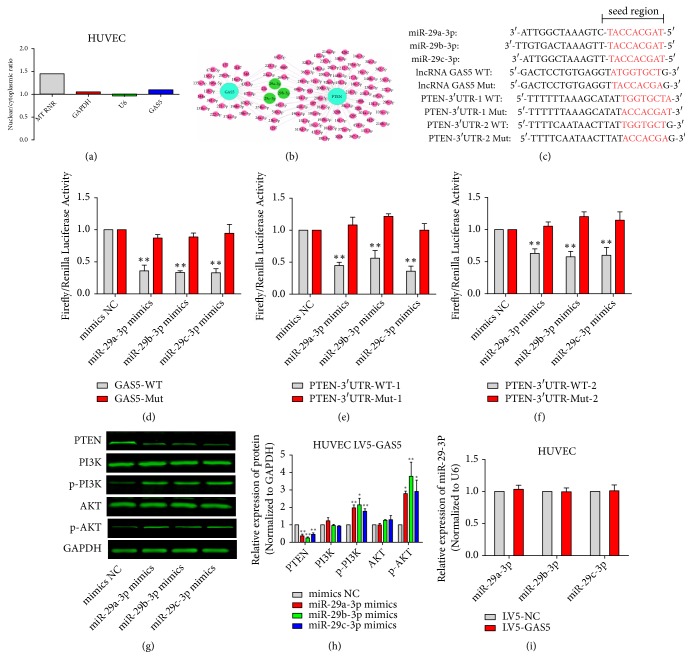
*GAS5 regulated PTEN, PI3K, and AKT by competitively binding miR-29-3p with PTEN.* (a) Cytoplasmic and nuclear GAS5 level in vascular HUVECs was detected by qPCR, MT RNR, GAPDH, and U6 served as the internal reference. (b) Cobinding miRNAs for GAS5 and PTEN were predicted by STARBASE 2.0 software. (c) The potential binding sites for GAS5 and PTEN-3′UTR in miR-29a-3p, miR-29b-3p, and miR-29c-3p. (d)–(f) Relative luciferase activity after 48 h cotransfection with the dual luciferase reporter constructs pmiRGlo-GAS5, pmiRGlo-PTEN-3′UTR-1, and pmiRGlo-PTEN-3′UTR-2 (wild-type or mutant vectors) and miR-29a-3p, miR-29b-3p, or miR-29c-3p mimics. NC mimics were used as control group. (g, h) Total PTEN, PI3K, AKT, phosphorylated p-PI3K, and p-AKT levels in the GAS5 overexpression group and GAS5 overexpression combined with cotransfection of miR-29a-3p, miR-29b-3p, or miR-29c-3p were detected by western blot. (i) The expression of miR-29a-3p, miR-29b-3p, and miR-29c-3p in the GAS5 overexpression HUVECs and control groups was detected by qPCR; U6 served as the internal reference. Statistical significance, *∗P*<0.05, *∗∗P*<0.01.

We used qPCR to detect cytoplasmic and nuclear GAS5 expression in HUVECs. GAS5 localization was analyzed by measuring the ratio of the CT values from the cytoplasm and nucleus; if the ratio was >1, it indicates that the tested RNA was predominantly located in the cytoplasm. MT RNR and GAPDH were the positive control for RNAs predominantly localized to the cytoplasm and U6 was the positive control for nuclear RNAs. These data showed that GAS5 was mainly located within the cytoplasm (Figure 6(a)). We used STARBASE 2.0 software to predict the potential miRNAs that could bind both GAS5 and PTEN (Figure 6(b)); among them, the target genes of miR-29-3p were significantly enriched in tumor-associated signaling pathways. We further conducted sequence comparisons of GAS5, the PTEN-3′UTR, and miR-29-3p (including miR-29a-3p, miR-29b-3p, and miR-29c-3p), finding that miR-29-3p had a binding site with GAS5 and had two binding sites with PTEN-3′UTR (Figure 6(c)). Next, we constructed pmiRGlo-GAS5, pmiRGlo-PTEN-3′UTR-1, and pmiRGlo-PTEN-3′UTR-2 (wild-type and mutant, respectively) vectors containing the binding sites of miR-29-3p. HEK293T cells were then cotransfected with the vectors and miR-29-3p mimics for 48 h, and relative luciferase activity was detected using the dual luciferase reporter assay. These results showed that miR-29-3p significantly reduced the relative luciferase activity of wild-type pmiRGlo-GAS5, pmiRGlo-PTEN-3′UTR-1, and pmiRGlo-PTEN-3′UTR-2, while mutant vectors did not show significant changes in luciferase activity (Figures 6(d)–6(f)). We further cotransfected miR-29a-3p, miR-29b-3p, and miR-29c-3p mimics into GAS5 overexpression HUVECs for 48 h. Western blot showed that, compared with the control group, PTEN protein expression in the transfected groups decreased significantly. PI3K and AKT protein levels showed no significant change; however, p-PI3K and p-AKT increased significantly (Figures 6(g) and 6(h)). Cotransfection of the miR-29a-3p, miR-29b-3p, and miR-29c-3p mimics and GAS5 offset the GAS5-induced increase in PTEN protein and decrease in p-PI3K and p-AKT. To further determine the effect of GAS5 on miR-29a-3p, miR-29b-3p, and miR-29c-3p, we used qPCR to detect miR-29a-3p, miR-29b-3p, and miR-29c-3p expression in the GAS5 overexpression HUVECs and control groups. However, we did not observe obvious changes in miR-29a-3p, miR-29b-3p, and miR-29c-3p levels following GAS5 overexpression (Figure 6(i)). In conclusion, these results suggested that GAS5 may act as an endogenous “sponge,” regulated PTEN, p-PI3K, and p-AKT by competitively binding miR-29-3p with PTEN, not by regulating the expression of miR-29-3p.

## 4. Discussion

Urethane is a class 2A carcinogen according to the International Agency for Research on Cancer. Previous studies have shown that urethane can specifically induce the development of lung adenocarcinoma in mice, and its pathologic characteristics are similar to those of human lung adenocarcinoma [12, 13]. We established a urethane-induced lung cancer animal model that was used to study the molecular mechanisms of lung cancer at different stages.

It has been found that tumor cells produce and release large amounts of exosomes into the microenvironment. These exosomes can affect the biological functions of adjacent cells or even cells in distant organs; they can also impact cancer progression. Zomer et al. fluorescently labeled living organisms and studied the associated biological behaviors using live imaging technology, finding that invasive cancer cells release exosomes that could be uptaken by low malignancy cancer cells, causing these cells to become more malignant and begin to metastasize. They also confirmed that exosomes could reach other organs through blood circulation to achieve a long-distance replication of malignant behaviors [14].

The functions of exosomes in oncogenesis and cancer development depend upon their origins and contained components. Noncoding RNAs, including miRNAs and lncRNAs, have been found to be enriched in exosomes; these noncoding RNAs may be involved in cancer development and/or progression [15, 16]. The roles of exosomal miRNAs in cancer have been deeply studied; however, exosomal lncRNAs have only been reported in a few studies. For example, Takahashi et al. reported that transforming growth factor-*β*1 induced enrichment of Linc-ROR in exosomes released by liver cancer cells and that this had important regulatory functions for chemotherapy sensitivity of liver cancer cells [17]. Li et al. found that LINC00152 levels in plasma exosomes from patients with gastric cancer and healthy controls were significantly different, suggesting LINC00152 is a potential biomarker for gastric cancer [18]. However, the functions and potential molecular mechanisms of lung cancer-associated exosomal lncRNAs have not been reported.

Through analyzing our previously published lncRNA chip data from urethane-induced mouse lung cancer tissues [19] as well as reviewing the related literature [7, 9, 20–24], we screened 19 lncRNAs for further analysis. This analysis showed that mouse serum exosomes contained various lncRNAs. Of note, GAS5 was significantly reduced in the serum exosomes from urethane-induced lung cancer mice and lung cancer tissues compared with control samples. Moreover, exosomal GAS5 levels and GAS5 expression in lung cancer tissues were positively correlated. The important functions of GAS5 in lung cancer have been identified in several studies. Tan et al. recently reported that, compared with healthy controls, plasma GAS5 levels in patients with nonsmall cell lung cancer were significantly decreased, but significantly increased after surgery [25]. Our study supports the importance of exosomal GAS5 in lung cancer.

We further detected GAS5 expression in human lung cancer tissue samples and lung cancer cell lines. GAS5 was expressed at low levels in lung cancer tissues and cell lines, which was consistent with the current literature. We also detected GAS5 levels in cell culture supernatant exosomes and found that exosomes from A549, H1299, and 95D lung cancer cells contained lower GAS5 level than normal 16HBE lung cells.

Angiogenesis is critical for cancer growth and metastasis. The positive rate of CD34 staining in 30 human lung cancer tissue samples was 83.3%, indicating that angiogenesis is common in lung cancer. Endothelial cells are an important interstitial cell type within the tumor microenvironment. These cells can exchange information with tumor cells through exosomes to further affect the formation of tumor neovasculature. We used exosomes from lung cancer cells to treat human umbilical vein endothelial cells (HUVECs) and demonstrated that these exosomes promoted HUVECs cell proliferation and tube formation and inhibited their apoptosis. New evidence shows that exosome-enriched lncRNAs may have special effects on cancer development; our data suggested that changing GAS5 content in tumor cell-derived exosomes affects the functions and phenotypes of HUVECs.

To study the function of exosomal miRNAs, previous studies have modulated exosomal miRNA levels by synthesizing mimic miRNA fragments and applying special transfection reagents and then observing changes in the host cell after transfection. This strategy is applicable for miRNAs, as they are short and stable; however, this approach is not appropriate for lncRNAs, which are longer, and chemically synthesized lncRNA fragments are prone to degradation. Additionally, the available technology and reagents for exosome transfection are immature. Therefore, we first used lentiviral-mediated GAS5 overexpression in lung cancer cells and then collected exosomes from cell culture supernatant. Although this method did not increase exosomal GAS5 levels as dramatically as the transfection of exosomes with GAS5 mimics, GAS5 levels in supernatant exosomes were significantly increased. We then treated HUVECs with these exosomes and found that exosomes from lung cancer cells with higher GAS5 content inhibited HUVECs proliferation and tube formation and promoted HUVECs apoptosis. These results suggested that GAS5 in exosomes from tumor cells could affect the functions and phenotypes of HUVECs.

LncRNAs regulate gene expression through various mechanisms and on multiple levels; however, the specific mechanisms of GAS5 on HUVECs were unknown. It has been reported that PTEN plays a key role in lung cancer angiogenesis [26, 27]. We found that PTEN mRNA was significantly reduced in human lung cancer tissue samples and was positively correlated with GAS5 expression. Therefore, we speculated that GAS5 may affect endothelial cell phenotypes by regulating PTEN. To test this hypothesis, we overexpressed GAS5 in HUVECs and found that PTEN mRNA and protein expression were increased by GAS5 overexpression. The close relationship between noncoding RNAs and mRNAs as well as the underlying interaction between them has become a hot topic in recent years [28]. Salmena et al. put forward competing endogenous RNAs (ceRNAs) hypothesis, which postulates that ceRNAs, including lncRNAs, mRNAs, and other RNA transcripts, act as sponges for miRNAs through their miRNA response elements (MREs), inhibiting the functions of their target miRNAs. Additionally, a MRE may be shared by various RNAs [29]. Studies have found that many lncRNAs, including lncRNA-ROR, MALAT1, and GAS5, can affect tumor development by acting as ceRNAs [30–32]. We detected GAS5 in the cytoplasm of HUVECs, and bioinformatics analyses identified that GAS5 and PTEN-3′UTR have common binding sites for certain miRNAs. Thus, we posit that GAS5 may play its roles through a ceRNA-like mechanism. The target genes of miR-29-3p (including miR-29a-3p, miR-29b-3p, and miR-29c-3p) are significantly enriched in tumor-related signaling pathways. Luciferase reporter assays also confirmed that miR-29-3p significantly attenuated the luciferase activity of the GAS5 and PTEN-3′UTR wild-type reporter vectors, suggesting that both GAS5 and PTEN-3′UTRs can directly bind miR-29-3p. Then we cotransfected miR-29-3p mimics into GAS5-overexpressing HUVECs and found that PTEN expression was inhibited by miR-29-3p mimics in the context of GAS5 overexpression. PTEN is suppressor gene for the PI3K/AKT pathway that inhibits PI3K/AKT activation. It has also been reported that PI3K/AKT pathway activation is involved in vascular endothelial cell function [33, 34]. Thus, we next detected levels of PI3K and AKT proteins and their phosphorylation status (p-PI3K and p-AKT). We found that, while PI3K and AKT protein expression levels were unchanged after GAS5 overexpression in HUVECs, p-PI3K and p-AKT were significantly reduced. Cotransfecting miR-29-3p mimics into HUVECs in which GAS5 was overexpressed significantly increased p-PI3K and p-AKT levels. To further determine the effect of GAS5 on miR-29-3p, we detected miR-29-3p expression in the GAS5 overexpression HUVECs and control groups. However, we did not observe obvious changes in miR-29-3p expression following GAS5 overexpression; these results suggested that GAS5 regulated PTEN by competitively binding miR-29-3p with PTEN, not by regulating the expression of miR-29-3p. The data presented here are consistent with recent reports supporting that lncRNAs act as “sponges” to bind specific miRNAs and regulate their function. For example, MIR31HG may function as endogenous decoy for miR-193b to affect its distribution on specific targets without inducing miR-193b destabilization, as the level of miR-193b was not affected following MIR31HG knockdown or overexpression [35]. Together, these results suggested that GAS5 can affect the PI3K/AKT signaling pathway to further modulate endothelial functions by regulating PTEN expression.

In conclusion, we reported, for the first time, that exosomal GAS5 participates in lung cancer angiogenesis. Our results support the potential of exosomal GAS5 to be a new target for inhibiting lung cancer angiogenesis. We hypothesize that the roles of GAS5 in the proliferation, apoptosis, and tube formation of HUVECs are fulfilled by competitively binding miR-29-3p with PTEN, and this model needs to be verified by in vivo studies.

## Data Availability

All data generated or analysed during this study are included in this published article; if necessary, the datasets used and/or analysed during the current study are available from the corresponding author upon reasonable request.

## Supplementary Materials

Supplementary Materials Supplementary Table 1 shows the sequences of the primers; GAPDH and U6 served as the internal reference. Supplementary Table 2 shows the sequences of the primers; GAPDH and U6 served as the internal reference. Supplementary Table 3 shows the sequences of the primers; Cel-miR-39-3p served as the external reference.
