# Supplementary material for: Low Long Noncoding RNA Growth Arrest-Specific Transcript 5 Expression in the Exosomes of Lung Cancer Cells Promotes Tumor Angiogenesis
Source: J Oncol. 2019 May 2;2019:2476175. doi: 10.1155/2019/2476175 (PMC6521541; doi:10.1155/2019/2476175)
Supplement: Supplementary Materials — Supplementary Table 1 shows the sequences of the primers; GAPDH and U6 served as the internal reference. Supplementary Table 2 shows the sequences of the primers; GAPDH and U6 served as the internal reference. Supplementary Table 3 shows the sequences of the primers; Cel-miR-39-3p served as the external reference. [file 2476175.f1.pdf]

Supplementary table 1

	Forward (5'-3')	Reverse (5'-3')
GAPDH	CAATGACCCCTTCATTGACC	GACAAGCTTCCCGTTCTCAG
U6	CTCGCTTCGGCAGCACA	AACGCTTCACGAATTTGCGT
AK016354	CTGGGTAATGGCTTGTTT	GATCTGATGCCCTCTTTT
AK041746	AATGAAACTAAAGAGGCACG	AGGGAGGACTATTTGGAGAA
AK169506	ACACCTTCAGTCCCATTT	TCTTTGGACCATCGTGAG
AK165804	GCTGAACAAATGATGGCGCT	AAACAGCACATGGCATCGG
AK006202	GACTCCATCAATCCCACC	CAACTGAAGCAACGGAAG
AK008754	AGTGGGAATGGATAAGACA	ACATAGGTGGAGCGAGAT
AK014679	GGGTTAGTGATTGTGGAG	ATGGCTGGGTATAGGTTA
AK017233	GATGCCTGGTTCCTTTAC	AGGGCTGTCTGCTATTTACT
AK030127	AAGACCCAGCCCAAAGAT	TGTGCTCGGAAGTCGTAT
AK086245	ATGGCACAACCGCAATGAAC	CAAGATCCCCGAGACGGATG
AK040806	CAACTGCAAGTGGGACTCCT	GCAGGCTAAGCCCATGGAAA
AK084832	CTGTGGGAGATGAGGTGG	GGAAGGCTGGTAGGAGGT
MALAT1	CTTGGCTTGTCAACTGCG	GTTGCCGACCTCAAGGAATG TTACC
HOTAIR	GAGCCAGAGCTGAAGGTATG	AAGACACGCACGGAGAAAGG
GAS5	GGAAGTACCTGGATTAGAG	ACTGAGTTACCTGGCTGT
LINCRNA -P21	CCCTGGACCTCATTACTT	AATTGCTCGTTCTTCTTATC
SOX2OT	ACCAGCACAAATGAACGG	CCAATGGCAAACCAAGTA
SNHG1	GCCCTTTACACTTTGGAG	CCTTTGAATGCCTGAGAT
TUG1	CAAGAAACAGCAACACCAGAA G	TAAGGTCCCCATTCAAGTCAG T

Real-time qPCR was used to detect the levels of lncRNAs in mouse serum exosomes and lung tissues, Supplementary table 1 shows the sequences of the primers, GAPDH and U6 served as the internal reference.

Supplementary table 2

	Forward (5'-3')	Reverse (5'-3')
GAPDH	CAATGACCCCTTCATTGACC	GACAAGCTTCCCGTTCTCAG
U6	TGCTCGCTTCGGCAGCACAT	CTTGCGCAGGGGCCATGCTA
GAS5	TGGTTCTGCTCCTGGTAA	GGTCTGCCTGCATTTCTT
MALAT1	AAGAAGCCGAAATAAATGAG	ACTGAAGCCCACAGGAAC
TUG1	CTCCTTATGCCGCCTTAGTG	TCCAAGGTTCCCAGGTTCA
PTEN	GGACGAACTGGTGTAATG	GCCTCTGACTGGGAATAG
MT RNR	CCTCCCCAATAAAGCTAAAA	GCTATTGTGTGTTCAGATAT

Real-time qPCR was used to detect the levels of lncRNAs in the human cells and lung tissues, Supplementary table 2 shows the sequences of the primers, GAPDH and U6 served as the internal reference.

Supplementary table 3

	Reverse-transcription primers (5'-3')	Forward primers (5'-3')	Consensus reverse primers (5'-3')
Cel-miR-39-3p	CTCAACTGGTGTCGTGGAGTCGGCAATTCAGTTGAGTGTCGTTC	ACACTCCAGCTGGGTCACCGGGTGTAAATC	TGGTGTCGTGGAGTCG
miR-29a-3p	CTCAACTGGTGTCGTGGAGTCGGCAATTCAGTTGAGTAACCGAT	ACACTCCAGCTGGGTAGCACCATCTGAAAT	TGGTGTCGTGGAGTCG
miR-29b-3p	CTCAACTGGTGTCGTGGAGTCGGCAATTCAGTTGAGAACACTGA	ACACTCCAGCTGGGTAGCACCATTTGAAATC	TGGTGTCGTGGAGTCG
miR-29c-3p	CTCAACTGGTGTCGTGGAGTCGGCAATTCAGTTGAGTAACCGAT	ACACTCCAGCTGGGTAGCACCATTTGAAAT	TGGTGTCGTGGAGTCG

Real-time qPCR was used to detect the levels of miRNA in serum exosomes or cells.

Supplementary table 3 shows the sequences of the primers, Cel-miR-39-3p served as the external reference.
